# The Direct and Indirect Effects of Methamphetamine on Fetuses and Infants

**DOI:** 10.3390/diagnostics16182962

**Published:** 2026-09-13

**Authors:** Ahmet Sedat Dündar, İsmail Altın, Yusuf Atan

**Affiliations:** 1 Bursa Forensic Medicine Group Directorate, Council of Forensic Medicine, Ministry of Justice, Bursa 16090, Türkiye; dr_asedat@hotmail.com; 2 Department of Forensic Medicine, School of Medicine, Istanbul Medeniyet Unıversity, Istanbul 34722, Türkiye; 3 Department of Forensic Medicine, Faculty of Medicine, Şeyh Edebali University, Bilecik 11230, Türkiye; dr.yusufatan@gmail.com; 4 Council of Forensic Medicine (Istanbul), Istanbul 34196, Türkiye

**Keywords:** pregnancy, methamphetamine, neonatal, autopsy, forensic medicine

## Abstract

**Background/Objectives**: Prenatal methamphetamine exposure may adversely affect fetal and neonatal outcomes, but its contribution to fetal and infant death remains incompletely understood. This study aimed to describe six fetal or infant deaths in which methamphetamine and amphetamine were detected and maternal methamphetamine exposure was considered a possible contributing factor. **Methods**: After obtaining the necessary approvals, medico-legal records, autopsy findings, and toxicological results were retrospectively reviewed for six cases from Bursa (*n* = 3) and Şanlıurfa (*n* = 3). **Results**: One case occurred in 2019, one in 2020, and four in 2021. Five cases involved preterm delivery between 24 and 32 gestational weeks, whereas Case 4 was born at 37 gestational weeks. Three cases were stillbirths, two infants died on postnatal day 4, and the term-born infant died on postnatal day 17. Methamphetamine and amphetamine were detected in postmortem specimens from all cases. Blood samples were available in five cases, with methamphetamine concentrations ranging from 19 to 256 ng/mL and amphetamine concentrations ranging from 4 to 42 ng/mL. In one case, only decomposition fluid was available. The organ damage attributable to methamphetamine and its role in the pathophysiology of death could not be fully established. Stimulant exposure was considered the most probable cause of death in Case 3, methamphetamine exposure was classified as the direct cause of death in Case 6, and methamphetamine exposure was considered a predisposing factor in the remaining four cases. **Conclusions**: Although methamphetamine and amphetamine were detected in all cases, these findings alone do not establish a causal relationship with death. Larger studies are needed to clarify prenatal and postnatal exposure pathways and to establish interpretive toxicological reference ranges for methamphetamine in fetal and infant populations.

## 1. Introduction

The use of narcotics and stimulants is an important societal problem in Türkiye, as it is throughout the world. The number of users of narcotics and stimulants has increased in recent years, and the mean age of users is decreasing [1,2].

According to the United Nations Office on Drugs and Crime, approximately 292 million people worldwide used drugs in 2022, representing a 20% increase over the preceding decade; amphetamines were used by an estimated 30 million people [3]. Methamphetamine use during pregnancy has also become an increasing public health concern. In highly affected populations, the estimated prevalence of methamphetamine use during pregnancy has ranged from 0.7% to 4.8% [4]. In the United States, the rate of amphetamine-related delivery hospitalizations, predominantly involving methamphetamine, doubled from 1.2 per 1000 deliveries in 2008–2009 to 2.4 per 1000 deliveries in 2014–2015 [5]. These trends indicate that prenatal methamphetamine exposure is an increasingly relevant maternal, neonatal, and forensic health issue.

Tobacco, primarily due to nicotine dependence, is the most commonly used addictive substance during pregnancy, followed by alcohol, cannabis and then methamphetamine, the use of which has increased globally in recent years [6]. Methamphetamine and its metabolites can be transmitted to an infant through maternal breast milk or contaminated surfaces with which the infant is in contact [7]. The use of methamphetamine in pregnancy has been reported to be associated with premature or caesarean birth, low Apgar score, and neonatal death [4,8]. Although signs of deprivation such as lethargy can be seen in the postpartum period, only 4% of affected infants require treatment. In infants exposed to methamphetamine through maternal milk, suckling problems, irritability, disrupted sleep patterns, agitation, and crying episodes may be seen [4,8]. Every substance with placental transmission can affect organ maturation, enzyme functions, and metabolic capacities of the fetus [9]. Consequently, the detection of methamphetamine or amphetamine in an infant postmortem specimen may not distinguish prenatal transplacental exposure from postnatal exposure through breast milk or other sources, particularly when paired maternal, placental, and breast-milk samples are unavailable [7]. However, validated toxic and fatal methamphetamine concentration ranges specific to fetuses and infants have not been established, which limits the interpretation of postmortem concentrations and prevents causality from being determined on the basis of concentration alone [10]. Toxicological interpretation is further complicated by postmortem redistribution, differences between specimen types, decomposition-related changes, and the immature hepatic and renal drug metabolism of fetuses and infants [10,11,12,13,14,15].

To our knowledge, this is one of the few studies from Türkiye to evaluate fetal and infant deaths associated with maternal methamphetamine exposure through the combined assessment of autopsy, histopathological, and toxicological findings. The aim of this study was to evaluate the potential indirect contribution of methamphetamine as a predisposing factor in fetal and infant deaths through forensic interpretation of the toxicological findings and autopsy results of six fetal and infant cases with detected methamphetamine and associated metabolites. It was also considered that the study will contribute to forensic decision-making by discussing the findings within a multidisciplinary perspective involving forensic medicine, pediatrics, and obstetrics, and thereby raise awareness of maternal substance use during pregnancy.

## 2. Materials and Methods

The study was conducted in strict accordance with the principles of the Declaration of Helsinki. Formal institutional and ethical approval for this research was granted by The Council of Forensic Medicine Scientific Research Committee (approval dated 10 May 2023, no. 21589509/2023/478, and dated 9 August 2023, no. 21589509/2023/754). Because the cases involved routine postmortem examinations legally mandated by judicial authorities, all procedures followed official forensic protocols. To maintain strict confidentiality and adhere to data protection regulations, all maternal and infant clinical data, demographic details, and forensic records were fully anonymized prior to evaluation, ensuring that no identifiable personal information was disclosed.

Between 2019 and 2021, 520 fetal and infant autopsies were performed at the two participating forensic medicine centers, including 418 cases in Şanlıurfa and 102 cases in Bursa. The available aggregated institutional records did not permit a reliable retrospective subdivision of these 520 cases into fetal deaths and postnatally deceased infants. All 520 cases underwent systematic toxicological screening, irrespective of the clinical history or investigative suspicion. Methamphetamine and/or amphetamine was detected in six cases, and all six positive cases were included in the study. No methamphetamine- or amphetamine-positive case was excluded. From this population, six fetal or infant cases with confirmed methamphetamine and/or amphetamine detection were identified and included in the study. Methamphetamine and/or its metabolites were detected through postmortem toxicological analyses of biological samples obtained during autopsy. These cases were compared in respect of the mother’s age, gestational week, sex, ethnicity, place of birth, and cause of death. The study cases were classified based on the role of methamphetamine in the cause of death. Methamphetamine was considered the direct cause of death when toxicological and autopsy findings indicated no other sufficient cause and its effects alone were pathophysiologically consistent with death. Stimulant exposure was considered the most probable cause of death when the available findings supported its causal contribution more strongly than other explanations, but evidentiary limitations prevented a definitive causal determination. In contrast, it was considered a predisposing factor when other primary causes were present, but methamphetamine may have contributed to or exacerbated the clinical condition.

In this study, fetus referred to an intrauterine case before live birth, stillbirth to fetal death with no signs of life at delivery, early neonatal death to the death of a live-born infant within the first seven completed days of life, and infant to a live-born child younger than one year. Gestational age was determined based on the available obstetric and hospital records.

### 2.1. Autopsy Examination

All the cases underwent complete forensic autopsy according to standard forensic protocols. The external examination included assessment of body measurements, developmental characteristics, congenital anomalies, signs of trauma, and evidence of medical intervention. The internal examination was performed through systematic evaluation of the cranial, thoracic, and abdominal cavities and their contents. Representative tissue samples from major organs, including the brain, cerebellum, brainstem, heart, lungs, liver, kidneys, spleen, pancreas, thymus, adrenal glands, intestines, and umbilical cord, were collected when available for histopathological examination. Toxicological analyses were performed using available postmortem biological samples, including cardiac blood, peripheral blood, urine, and decomposition fluid, depending on sample availability and preservation status. The autopsy, histopathological, and toxicological findings were evaluated together to determine the cause of death and the potential contribution of methamphetamine exposure.

The classification of methamphetamine as a direct cause of death or a predisposing factor was based on the final forensic reports issued by the responsible forensic committee after the toxicological results became available. The committee evaluated the circumstances of death together with the autopsy, histopathological, and toxicological findings.

Body length and weight measurements were obtained during the postmortem examination. The sex-specific 2013 Fenton growth chart was used to evaluate the preterm cases according to gestational age in stillbirth cases and postmenstrual age at death in live-born cases [16]. Postmenstrual age was calculated by adding postnatal age to gestational age at birth. Case 4, who was born at 37 gestational weeks and died on postnatal day 17, was evaluated according to the sex- and age-specific World Health Organization Child Growth Standards [17]. In the live-born cases, the resulting percentiles represent body size at the time of death and were not interpreted as birth-size classifications. Because birth length and weight measurements were unavailable for these infants, their small for gestational age (SGA), appropriate for gestational age (AGA) or large for gestational age (LGA) status at birth could not be determined. For the stillbirth cases, SGA was defined as a body weight below the 10th percentile for sex and gestational age, AGA as a body weight between the 10th and 90th percentiles, and LGA as a body weight above the 90th percentile.

### 2.2. Histopathological Examination

Histopathological examination was performed solely to evaluate organ pathology and was not used to determine the presence of methamphetamine or amphetamine. Representative samples from the brain, cerebellum, brainstem, heart, lungs, liver, kidneys, spleen, pancreas, thymus, adrenal glands, intestines, umbilical cord, and placenta, when available, were fixed in 10% neutral-buffered formalin, routinely processed, stained with hematoxylin and eosin, and examined by light microscopy. The histopathological findings were evaluated jointly by an experienced forensic medicine specialist and a pathologist. The hematoxylin–eosin-stained sections provided sufficient morphological findings for histopathological assessment, and no diagnostic uncertainty requiring additional histochemical or immunohistochemical staining was identified. Therefore, no additional staining was performed in the present cases.

### 2.3. Sample Collection and Toxicological Analysis

Samples were collected during autopsy. Cardiac blood was obtained in four cases, both cardiac and peripheral blood in one case, and only decomposition fluid in one case due to advanced decomposition. All the samples were placed in EDTA tubes and transported under cold chain conditions (2–8 °C) to the laboratory. Upon arrival, blood and urine samples were stored at −20 °C until toxicological analysis. None of the samples were collected specifically for this study.

All the cases underwent systematic postmortem toxicological screening for alcohol, abused substances, and therapeutic drugs as part of routine forensic investigative procedures. Initial screening was performed using established forensic toxicological methods, including headspace gas chromatography with flame ionization detection (GC-FID) for volatile compounds, gas chromatography–mass spectrometry (GC-MS), liquid chromatography–tandem mass spectrometry (LC-MS/MS), and liquid chromatography–high-resolution mass spectrometry (LC-HRMS), depending on sample availability and condition. Cases were flagged as positive for methamphetamine and/or amphetamine based on qualitative detection during screening analyses.

In routine forensic practice, cases that screened positive for methamphetamine and/or amphetamine were subjected to confirmatory and quantitative analysis when sufficient sample volume and quality were available. Quantitative analyses were performed as part of routine investigative procedures and were not conducted retrospectively as a batch analysis. In cases with advanced decomposition or limited biological material, qualitative detection without reliable quantification was reported.

Quantitative confirmation was performed using a validated LC-MS/MS method. Solid-phase extraction (SPE) was carried out using OASIS HLB 3 cc (60 mg) cartridges. Briefly, 0.5 mL of blood was diluted with 2 mL of distilled water, vortexed, and centrifuged at 5000 rpm for 10 min. Calibrators and quality control samples were prepared by spiking blank blood samples with appropriate working solutions. The calibration range was 0.5–100 ng/mL, and the quality control concentration was 20 ng/mL.

The SPE cartridges were conditioned with 2 mL ethyl acetate, 2 mL methanol, and 2 mL distilled water. After sample loading, the cartridges were washed with 2 mL of 5% methanol in water (*v*/*v*) and dried under a nitrogen stream for 20 min. Elution was performed with 2 × 0.5 mL methanol followed by 2 × 0.5 mL ethyl acetate. The eluate was evaporated to dryness at 40 °C under nitrogen and reconstituted in 0.5 mL methanol:water (20:80, *v*/*v*) prior to analysis.

UHPLC-MS/MS analysis was conducted using an ultra-high-performance liquid chromatography–tandem mass spectrometry system consisting of a Shimadzu Nexera X2 LC-30AD UHPLC system coupled to a Shimadzu 8050 triple quadrupole mass spectrometer (Shimadzu, Kyoto, Japan). Chromatographic separation was achieved using an Agilent Poroshell 120 EC-C18 column (150 × 4.6 mm, 2.7 µm). The mobile phase consisted of 5 mM ammonium acetate containing 0.1% formic acid in water (mobile phase A) and methanol (mobile phase B). A 15 min gradient elution was applied, starting at 10% B (0–0.3 min), increasing to 80% B (0.3–3 min), then to 95% B (3–7 min), and held at 95% B for 4 min (7–11 min), followed by re-equilibration to 10% B (11–15 min). The column temperature was maintained at 40 °C, with a flow rate of 0.6 mL/min.

Detection was performed in positive electrospray ionization mode using multiple reaction monitoring (MRM). The precursor ion for methamphetamine was *m*/*z* 150.15 with product ions at *m*/*z* 91.10 and 119.15 (retention time 5.06 min), and for amphetamine, *m*/*z* 136.10 with product ions at *m*/*z* 91.10 and 119.15 (retention time 5.10 min).

Method validation was performed in accordance with the Scientific Working Group for Forensic Toxicology (SWGTOX) guidelines [18]. Linearity was assessed using matrix-matched calibration curves over the concentration range of 0.5–100 ng/mL. Limits of detection (LOD) and limits of quantification (LOQ) were determined based on signal-to-noise ratios of 3:1 and 10:1, respectively. Recovery was assessed by comparing extracted samples with post-extraction spiked controls. The 0.5 ng/mL concentration represented the lowest calibration standard, whereas quantitative reporting was based on the analyte-specific LOQ values.

The validation parameters of the analytical method, including LOD, LOQ, recovery, and retention times for methamphetamine and amphetamine, are summarized in Table 1.

## 3. Results

Between January 2019 and December 2021, a total of 6 cases were detected, including 3 cases in Bursa Forensic Medicine Group Directorate and 3 cases in Şanlıurfa Forensic Medicine Branch Directorate. 1 case belonged to 2019, 1 case belonged to 2020, and 4 cases belonged to 2021. The youngest mother was 19 years old and the oldest was 32 years old. Five infants were delivered preterm between 24 and 32 gestational weeks, whereas Case 4 was delivered at 37 gestational weeks. 3 of the cases were stillbirths. Two of the cases were 4 days old and one was 17 days old. 4 of the cases were female and 2 were male. 4 of the cases were born in the hospital and 2 of them were born at home. In two cases, the mothers reportedly used medications in an attempt to terminate the pregnancy. Case 4 was reportedly breastfed. However, the available documentation did not indicate whether the mother used methamphetamine during the infant’s hospitalization, and no maternal or breast-milk toxicological analysis was available. Therefore, postnatal exposure through breast milk was considered possible but could not be confirmed, and prenatal transplacental exposure could not be excluded. In this case, postnatal exposure through breast milk was considered possible in addition to prenatal transplacental exposure; in the remaining cases, transplacental exposure was considered the most plausible route. Five of the six cases were born preterm. According to the Fenton growth reference, the three stillbirth cases were not classified as small for gestational age. In the live-born cases, length and weight measurements were obtained at the time of death rather than at birth. Therefore, these measurements were interpreted as body size at death, and SGA, AGA, or LGA status at birth could not be determined. In Case 4, who was born at 37 gestational weeks and died on postnatal day 17, both length and weight were below the 1st percentile according to the WHO Child Growth Standards. Recorded body lengths ranged from 31 to 47 cm, whereas body weights ranged from 588 to 2185 g. It was observed that the foramen ovale was still open in three cases, the ductus arteriosus was not closed in one case, and there was an atrial septal defect in one case. Apart from these findings, external and internal gross examinations revealed no additional traumatic lesion, lethal congenital anomaly, or other macroscopic pathological finding sufficient to explain death or stillbirth. Histopathological examination revealed bronchopulmonary dysplasia in Case 2, while the other pulmonary findings included congestion, pneumonia, bronchiolitis, aspiration-related changes, emphysematous changes, autolysis, and immaturity. Pulmonary hemorrhagic findings were observed in Cases 2, 4, and 5. Diffuse intra-alveolar hemorrhage was present in Cases 2 and 4, whereas mild erythrocyte extravasation in the subpleural, interstitial, and alveolar regions was observed in Case 5. Findings described as organ immaturity in the extremely preterm cases were considered compatible with their gestational age and were not interpreted as drug-specific effects. Similarly, patent foramen ovale and patent ductus arteriosus in the preterm cases were considered developmentally appropriate findings rather than structural abnormalities attributable to methamphetamine exposure. Detailed records regarding delivery-room resuscitation, chest compressions, endotracheal intubation, mechanical ventilation, and other advanced neonatal interventions were unavailable for Cases 2 and 5, both of whom died after four days of hospitalization. No resuscitative or advanced medical intervention was documented for Case 4. Therefore, a possible contribution of medical interventions to some pulmonary findings, particularly intraalveolar hemorrhage, could not be definitively assessed. Methamphetamine and amphetamine were quantitatively detected in blood samples from five cases, whereas both substances were qualitatively detected in decomposition fluid in Case 3. Stimulant exposure was considered the most probable cause of death in Case 3, methamphetamine exposure was classified as the direct cause of death in Case 6, and methamphetamine exposure was considered a predisposing factor in the remaining four cases (Table 2 and Table 3). Detailed histopathological findings, including immaturity and aspiration-related changes, are presented in Table 4.

## 4. Discussion

The present study provides a forensic evaluation of fetal and infant deaths associated with methamphetamine exposure in Türkiye by integrating autopsy, histopathological, and toxicological findings. The findings illustrate the complexity of interpreting methamphetamine exposure in fetal and infant deaths and suggest that, depending on the clinical and forensic circumstances, it may act either as a direct cause of death or as a predisposing factor. Methamphetamine use among women of childbearing age has increased worldwide and now exceeds the use of cocaine and other stimulants [19]. Recent reviews have associated prenatal methamphetamine exposure with fetal growth restriction, low birth weight, preterm birth, and adverse neurodevelopmental outcomes [20,21]. A large contemporary cohort study reported that prenatal methamphetamine use was associated with increased risks of preterm birth (aRR, 2.85) and infant death (aRR, 2.73) [22]. Another study found significantly higher rates of preterm birth and lower mean birth weight among methamphetamine-exposed pregnancies than among controls [23]. Consistent with these reports, prematurity was a prominent finding in the present series, occurring in five of the six cases. Collectively, these findings add to the growing body of evidence that maternal methamphetamine exposure may adversely affect fetal growth and perinatal outcomes.

Methamphetamine readily crosses the placenta and can directly expose the fetus. Perinatal amphetamine exposure has been associated with adverse pregnancy and developmental outcomes, although interpretation is complicated by coexisting medical, psychosocial, and polysubstance-related factors [24,25]. In their eight-case series, Stewart and Meeker reported methamphetamine intoxication as the cause of death in one case and considered maternal methamphetamine use relevant to death in four additional cases [26]. A separate report of concurrent maternal and fetal death due to fentanyl, methamphetamine, and cocaine toxicity further emphasized the importance of integrating the circumstances of death, autopsy findings, histopathology, and comprehensive toxicological analysis when evaluating substance-related deaths [27]. In the present study, intrauterine death occurred in three cases, and methamphetamine concentrations were higher than the corresponding amphetamine concentrations in all three. In two cases, death was attributed to maternal illicit substance use. However, age and organ maturity may influence drug toxicity, and established pediatric toxic and lethal concentration ranges remain limited; therefore, postmortem methamphetamine concentrations cannot be interpreted in isolation [28]. Accordingly, the distinction between a direct cause of death and a predisposing factor was based on the overall forensic evaluation of each case. Methamphetamine was considered a direct cause of death only when no sufficient alternative cause was identified and the combined circumstantial, autopsy, histopathological, and toxicological findings supported a potentially fatal contribution. In cases classified as involving a predisposing factor, methamphetamine was not regarded as the primary cause of death but as a potential contributor to adverse fetal or neonatal outcomes through mechanisms such as growth restriction, prematurity, and developmental vulnerability [24,29]. In Case 1, severe maternal trauma was considered the principal lethal event associated with intrauterine death, whereas methamphetamine exposure was classified as a predisposing factor because of its potential contribution to prematurity and developmental vulnerability. These findings indicate that methamphetamine-associated fetal and infant deaths may occur through different mechanisms and should be interpreted within the overall clinical, toxicological, and forensic context rather than on toxicological concentrations alone [27,28].

Many drugs can enter human milk, predominantly through passive diffusion, and the extent of transfer is influenced by factors including molecular weight, lipophilicity, ionization, plasma protein binding, and maternal plasma concentration [30]. Methamphetamine and amphetamine have been detected in breast milk and infant serum following maternal use, confirming that breastfeeding may constitute a potential route of postnatal exposure [7,30,31]. In a case involving prescribed racemic amphetamine, the mean milk-to-maternal plasma concentration ratio was approximately 3; however, findings obtained from therapeutic amphetamine exposure should not be directly extrapolated to illicit methamphetamine use [32]. Kenneally and Byard detected methamphetamine in the gastric contents of two deceased children and in prepared infant formula in one case, demonstrating potential postnatal routes of exposure other than transplacental transfer [10]. In the present series, only Case 4 was documented as having been breastfed. The available hospital records did not document maternal methamphetamine use during the infant’s hospitalization. Furthermore, the detection of methamphetamine in the infant on postnatal day 17 does not establish that the mother used methamphetamine in the hospital or that exposure occurred through breastfeeding. This infant survived for 17 days after birth, and the methamphetamine concentration was higher than the amphetamine concentration. Therefore, both prenatal transplacental exposure and postnatal exposure through breast milk were considered possible. However, this concentration relationship alone cannot establish the route or timing of exposure. In the three fetal deaths, transplacental transfer was considered the only plausible route because postnatal exposure was not possible. Maternal blood, breast milk, and placental toxicological analyses were unavailable; therefore, the relative contributions of prenatal and postnatal exposure in Case 4 could not be determined. Information on the timing and frequency of breastfeeding, maternal contact after delivery, and antemortem toxicological testing was unavailable. Accordingly, the routes of exposure in this series should be regarded as presumed rather than confirmed and interpreted according to the timing of death, available clinical history, autopsy findings, and toxicological results. The mechanisms through which methamphetamine may contribute to fetal and infant death remain incompletely understood, and further multidisciplinary studies incorporating paired maternal, placental, breast milk, and infant samples are needed.

In Case 3, a conventional blood sample could not be obtained because of advanced decomposition and the limited available blood volume in this extremely preterm fetus; therefore, toxicological analyses were performed on decomposition fluid, in which methamphetamine and amphetamine were detected. In markedly decomposed bodies, alternative matrices may provide useful evidence of prior drug exposure when conventional blood samples are unavailable [12,13,14,15]. However, toxicological findings obtained from non-standard matrices require cautious interpretation because decomposition, matrix effects, sampling site, and postmortem redistribution may affect drug detectability and measured concentrations [12,13,14,15,16]. Alasmari et al. reported that methamphetamine and amphetamine remained detectable in putrefied tissues and found no statistically significant difference in their concentrations between putrefied and non-putrefied cases; nevertheless, the authors emphasized multiple-matrix analysis and case-specific interpretation [12]. Although decomposition and autolytic changes were present, they did not preclude a complete external and internal autopsy. No sufficient traumatic, congenital, infectious, or other pathological cause capable of explaining the intrauterine death was identified. Methamphetamine and amphetamine detected in decomposition fluid confirmed fetal exposure; however, because this matrix was not validated for quantitative or semi-quantitative interpretation, neither a blood-equivalent concentration nor a reliable classification of the detected levels as residual or potentially toxic could be provided. Placental tissue was unavailable for histopathological examination, and an unrecognized placental disorder could therefore not be completely excluded. Accordingly, following the integrated evaluation of the circumstances, autopsy findings, available histopathological findings, and toxicological results, stimulant exposure was considered the most probable cause of death in Case 3, although a direct causal relationship could not be established with certainty. In Case 6, the methamphetamine concentration exceeded the validated upper limit of quantification (>100 ng/mL; reported as 119 ng/mL in the original toxicology report). However, because no validated fatal methamphetamine concentration has been established for fetuses, this finding was not considered independently diagnostic of fatal intoxication. Complete autopsy revealed a large atrial septal defect; however, this abnormality was not considered sufficient to explain the intrauterine death. Placental histopathology showed congestion, autolytic changes, and immaturity, without a specific inflammatory or other placental lesion sufficient to account for death, although the autolytic changes limited definitive placental assessment. No histopathological evidence of infection was identified in the available fetal tissues. Detailed maternal obstetric and clinical information was unavailable. Following the integrated evaluation of the circumstances, autopsy, available placental and fetal histopathology, and toxicological findings, and in the absence of another sufficient cause, stimulant exposure was retained as the direct cause of death as a diagnosis of exclusion rather than on the basis of the reported concentration alone.

Evidence directly linking methamphetamine exposure to death in fetuses, infants, or children remains limited and is derived primarily from case reports and retrospective forensic series rather than controlled experimental or prospective human studies [26,28,33]. In a retrospective review of 50 deaths among children aged ≤12 years in whom methamphetamine was detected in blood, urine, and/or hair, 66% were infants and the cause of death remained unascertained in 62% of cases. These findings demonstrate that the detection of methamphetamine exposure does not, by itself, establish a causal role in death [33]. Nevertheless, clinical studies have associated prenatal methamphetamine use with fetal growth restriction, low birth weight, preterm birth, and other adverse neonatal outcomes [22,23]. Experimental animal research has also demonstrated that prenatal exposure may impair fetal growth and glucose metabolism [34], while human neuroimaging studies have identified structural, metabolic, and functional brain alterations in prenatally exposed children [35]. More broadly, methamphetamine-induced neuronal injury has been associated with oxidative stress, neuroinflammation, excitotoxicity, mitochondrial dysfunction, DNA damage, and apoptotic cell-death pathways [36]. In the present series, five of the six cases were born preterm, and methamphetamine exposure was classified as either a direct cause of death or a predisposing factor based on the overall forensic evaluation. However, the available evidence does not permit these experimental and neurodevelopmental mechanisms to be directly extrapolated to lethal toxicity in fetal and infant cases. Therefore, the specific pathophysiological pathways through which methamphetamine exposure may contribute to fetal or infant death remain uncertain and require further investigation. The interval between delivery and postmortem sampling is important when interpreting toxicological findings in hospitalized newborns. Neonatal urine primarily reflects recent exposure, and amphetamines are generally reported to be cleared from neonatal urine within approximately 1–3 days after birth; however, this estimate cannot be directly applied to postmortem blood, particularly in extremely preterm infants [37,38]. The persistence of methamphetamine and amphetamine may be influenced by the timing and magnitude of maternal use before delivery, fetal drug accumulation, gestational age, immature hepatic and renal clearance, urinary pH, analytical sensitivity, specimen type, and postmortem redistribution. Experimental evidence also suggests that fetal elimination may be slower than maternal elimination, although corresponding human neonatal pharmacokinetic data remain limited [39]. In Cases 2 and 5, methamphetamine and amphetamine were detected in cardiac blood after four days of hospitalization, and no postnatal methamphetamine exposure was documented. These findings may therefore represent persistence of prenatal exposure, but they cannot establish the precise timing or magnitude of the last exposure. For forensic practice, postmortem toxicological findings in hospitalized newborns should be interpreted according to the interval since delivery and, where available, through analysis of multiple complementary matrices, because blood and urine primarily reflect relatively recent exposure, whereas meconium, umbilical cord tissue, and neonatal hair may document a longer period of prenatal exposure [37,38].

Prenatal methamphetamine exposure has been associated with impaired fetal growth in previous studies. In a recent Turkish autopsy-based study including 28 fetal deaths associated with prenatal methamphetamine exposure, 57.1% of the cases were classified as small for gestational age [40]. In the present series, however, the three stillbirth cases were not classified as SGA according to the Fenton growth reference. For the live-born infants, length and weight were measured at the time of death rather than at birth; therefore, their SGA status at birth could not be determined. Notably, in Case 4, who was born at 37 gestational weeks and died on postnatal day 17, both length and weight were below the 1st percentile according to the WHO Child Growth Standards. This finding indicates markedly low body size at the time of death but cannot, in the absence of birth measurements, establish fetal growth restriction or SGA status at birth. Differences between the present series and the previous Turkish study may be related to the small sample size, differences in case composition, and the unavailability of birth anthropometric data for the live-born infants.

### Limitations

The main limitation of this study was the absence of systematic placental histopathological examination in all cases. Placental findings may provide important information regarding fetal growth restriction, placental insufficiency, vascular pathology, and the effects of maternal substance use during pregnancy. As placental examination was not available for all cases, the potential contribution of placental pathology to the observed fetal and infant outcomes could not be fully evaluated.

Detailed records regarding resuscitation, endotracheal intubation, mechanical ventilation, and other advanced neonatal interventions were unavailable for Cases 2 and 5. Therefore, the potential contribution of these procedures to pulmonary findings, particularly intraalveolar hemorrhage, could not be determined.

Another limitation was that peripheral blood was available for toxicological analysis in only one case, whereas cardiac blood or other postmortem specimens were used in the remaining cases. This limitation restricts direct comparison between cases and may affect the interpretation of postmortem drug concentrations. Therefore, toxicological findings should be interpreted with caution.

Information on potential maternal and environmental confounders, including tobacco and alcohol use, other illicit substance use, nutritional status, prenatal care, maternal infection, and socioeconomic circumstances, was incomplete or unavailable. Therefore, prematurity, low body size, or other adverse outcomes in individual cases cannot be attributed specifically to methamphetamine exposure.

Detailed antenatal follow-up records and maternal gynecological and obstetric histories, including gravidity, parity, previous miscarriages or stillbirths, ultrasonographic findings, and pregnancy-related complications, were unavailable in the reviewed forensic records. This limitation prevented a comprehensive assessment of alternative maternal or pregnancy-related factors that may have contributed to stillbirth or preterm delivery.

The absence of complete placental, maternal, umbilical cord, and genetic or chromosomal data for all fetal cases limited the comprehensive exclusion of alternative causes of fetal death.

The small sample size and absence of a control group precluded statistical comparison and limit the generalizability of the findings. Because this was a retrospective forensic autopsy series conducted at two regional centers, the findings may also be affected by referral and selection bias and may not represent all methamphetamine-exposed fetal or infant deaths.

Gestational age was obtained from the available obstetric and hospital records but could not be independently verified in every case. In addition, the absence of birth anthropometric measurements in the live-born infants limited the assessment of fetal growth and SGA status. Decomposition and the use of nonstandard decomposition fluid in Case 3 further restricted toxicological and histopathological interpretation. Finally, although five of the present cases could be definitively distinguished from the previously published Turkish series of 28 fetal deaths, possible overlap involving one case could not be completely excluded because case-level identifiers were unavailable in the published report.

Autolytic changes in four cases, including advanced decomposition in Case 3, limited histopathological interpretation because such changes may obscure, mimic, or alter morphological findings; therefore, the nonspecific histopathological findings were interpreted cautiously.

Chiral analysis was not performed; therefore, the enantiomeric origin of methamphetamine could not be analytically confirmed. However, no prescription or other pharmaceutical source that could account for the findings was documented in the available medical and investigative records.

## 5. Conclusions

Methamphetamine exposure may contribute to adverse fetal and infant outcomes, including prematurity and death. In this series, stimulant exposure was considered the most probable cause of death in Case 3, methamphetamine exposure was classified as the direct cause of death in Case 6, and methamphetamine exposure was considered a predisposing factor in the remaining four cases based on the overall clinical, toxicological, histopathological, and forensic evaluation. These findings highlight the complexity of assessing methamphetamine-related deaths in fetuses and infants and emphasize the importance of multidisciplinary interpretation. From a forensic diagnostic perspective, methamphetamine-associated fetal and infant deaths should be evaluated through the integrated interpretation of the circumstances of death, specimen characteristics, toxicological results, autopsy findings, and histopathological findings rather than on toxicological concentrations alone. Further studies are needed to better clarify the mechanisms of maternal–fetal and postnatal transmission and the role of methamphetamine in fetal and infant mortality.

## Figures and Tables

**Table 1:** Validation parameters of the LC–MS/MS method for methamphetamine and amphetamine analysis.

	LOD (ng/mL)	LOQ (ng/mL)	Recovery (%)	Retention Time (min)	*m*/*z*
MA	0.56	0.66	98.5	5.06	150.15> 91.10
A	0.60	0.64	104.6	5.10	136.10> 91.10

MA: methamphetamine; A: amphetamine. LOD: limit of detection; LOQ: limit of quantification. MA: Methamphetamine 10 mg (d5-chiron AS)/A: Amphetamine 1000 ppm in 1mL methanol (d5-chiron AS).

**Table 2:** Demographic and Perinatal Characteristics of the Cases.

Case	Year	Maternal Age (Years)	Sex	Gestational Age/Postnatal Survival	Place of Delivery	Presumed Route of Exposure	Length (Percentile)	Weight (Percentile)	Growth Interpretation
1	2019	19	Female	32 gestational weeks (stillbirth)	Hospital	Transplacental	42 cm (50th–90th)	1729 g (50th–90th)	AGA
2	2020	29	Female	24 gestational weeks; survived for 4 days	Hospital	Transplacental	32 cm (50th–90th)	588 g (10th–50th)	Percentiles at death; birth-size classification unavailable
3	2021	19	Female	27 gestational weeks (stillbirth)	Home	Transplacental	37 cm (10th–50th)	1050 g (10th–50th)	AGA
4	2021	22	Male	37 gestational weeks; survived for 17 days	Hospital	Transplacental and possible exposure through breast milk	47 cm (<1st)	2185 g (<1st)	Low length- and weight-for-age at death; birth-size classification unavailable
5	2021	24	Female	24 gestational weeks; survived for 4 days	Hospital	Transplacental	32 cm (50th–90th)	810 g (>90th)	Percentiles at death; birth-size classification unavailable
6	2021	32	Male	25 gestational weeks (stillbirth)	Home	Transplacental	31 cm (10th–50th)	687 g (10th–50th)	AGA

Note: Length and weight were measured during the postmortem examination. For the preterm cases, percentiles were determined using the sex-specific 2013 Fenton growth chart according to gestational age in stillbirths and postmenstrual age at death in live-born infants. Case 4 was evaluated at postnatal day 17 using the sex- and age-specific WHO Child Growth Standards. AGA classification was assigned only to stillbirth cases, for whom the measurements reflected size at birth. In live-born cases, the percentiles describe body size at death and do not represent birth-size classifications. AGA, appropriate for gestational age.

**Table 3:** Exposure, Toxicological, and Medico-Legal Characteristics of the Cases.

Case	Analyzed Specimen	Toxicological Findings	Main Gross Autopsy Findings	Cause of Death	Role in Death
1	Cardiac blood	>100 ng/mL methamphetamine (reported as 256 ng/mL in the original toxicology report), 42 ng/mL amphetamine	Patent foramen ovale; no other significant gross finding	Intrauterine death as a result of maternal pelvis fractures due to traffic accident	Predisposing factor
2	Cardiac blood	28 ng/mL methamphetamine, 12 ng/mL amphetamine, 6 ng/mL fentanyl, 598,600 ng/mL lidocaine, 2 ng/mL prilocaine, 2400 ng/mL fluconazole, 62 ng/mL midazolam	No significant gross findings	Premature birth and its complications	Predisposing factor
3	Decomposition fluid	Qualitatively positive for methamphetamine, amphetamine, and paracetamol	Advanced decomposition; Patent foramen ovale, Patent ductus arteriosus	Intrauterine death stimulant exposure considered the most probable cause	Most probable cause
4	Cardiac blood	36 ng/mL methamphetamine, 13 ng/mL amphetamine	No significant gross finding	Respiratory failure due to lung infection	Predisposing factor
5	Cardiac blood and urine	19 ng/mL methamphetamine, 4 ng/mL amphetamine, 6 ng/mL fentanyl, 12,000 ng/mL paracetamol, 26 ng/mL midazolam, 18 ng/mL alpha-hydroxymidazolam	Patent foramen ovale; no other significant gross findings	Premature birth and its complications	Predisposing factor
6	Cardiac blood	>100 ng/mL methamphetamine (reported as 119 ng/mL in the original toxicology report), 6 ng/mL amphetamine	Large atrial septal defect; no other significant gross finding	Intrauterine death due to stimulants	Direct cause

Note: Patent foramen ovale and patent ductus arteriosus in the preterm cases were considered developmentally appropriate findings for gestational age and were not interpreted as congenital abnormalities or specific effects of methamphetamine exposure. The large atrial septal defect identified in Case 6 was considered a structural cardiac abnormality. Methamphetamine concentrations exceeding the validated upper limit of quantification (100 ng/mL) are presented as >100 ng/mL. The values shown in parentheses were recorded in the original toxicology reports but were outside the validated calibration range.

**Table 4:** Histopathological Findings.

**Panel A.** Central nervous system and major thoracoabdominal organs							
**Case**	**Brain, Cerebellum, and Brainstem**	**Heart**	**Lungs**			**Liver**	**Kidneys**
1	Autolytic changes in glial tissues	Congestion	Congestion in the interstitium, erythrocyte extravasation, pink squamous structures in the alveoli			Congestion, extramedullary hematopoiesis	Congestion
2	Hemorrhage in the ventricular cavities and periventricular brain parenchyma, congestion	congestion	Fibroblast proliferation and thickening with fibrosis in the interalveolar septum, type II pneumocyte hyperplasia and presence of hyaline membrane remnants and fibrin in the alveoli, bronchopulmonary dysplasia, diffuse intraalveolar hemorrhage, congestion			Mild mononuclear inflammatory cell infiltration and fibrosis-related expansion in portal areas, widespread extramedullary hematopoiesis, congestion	Congestion
3	Autolysis	Severe autolysis	Amnion and meconium aspiration, autolysis, congestion			Severe autolysis	Severe autolysis
4	Congestion	Congestion	Intraalveolar diffuse hemorrhage and few histiocytes, hemorrhage in the pleura, interlobular area and septum, lobular pneumonia, purulent bronchiolitis, few amnions in the alveolar lumens			Congestion	Congestion
5	Extravasated erythrocytes in the subarachnoid space, congestion, autolytic changes and immaturity	Congestion, autolytic changes and immaturity	Emphysematous changes, mild erythrocyte extravasation in the subpleura, interstitium and alveolar lumens, congestion, autolytic changes and immaturity			Congestion, autolytic changes and immaturity	Congestion, autolytic changes and immaturity
6	Congestion, autolytic changes and immaturity	Congestion, autolytic changes and immaturity	Congestion, autolytic changes and immaturity			Congestion, autolytic changes and immaturity	Congestion, autolytic changes and immaturity
**Panel B.** Other organs and tissues							
**Case**	**Spleen**	**Pancreas**	**Thymus**	**Placenta**	**Adrenal Glands**	**Intestine**	**Umbilical Cord**
1	Congestion	Congestion	Congestion	Could not be sampled	Autolytic changes and congestion	Could not be sampled	Two arteries and one vein; no pathological findings
2	Could not be sampled	Could not be sampled	Congestion	Could not be sampled	Congestion	Congestion	Could not be sampled
3	Severe autolysis	Could not be sampled	Autolysis, congestion	Could not be sampled	Severe autolysis	Severe autolysis	Two arteries and one vein; autolysis
4	Congestion	Could not be sampled	Congestion	Could not be sampled	Congestion	Autolysis	Could not be sampled
5	Congestion, autolytic changes and immaturity	Could not be sampled	Could not be sampled	Could not be sampled	Congestion, autolytic changes and immaturity	Could not be sampled	Could not be sampled
6	Congestion, autolytic changes and immaturity	Congestion, autolytic changes and immaturity	Congestion, autolytic changes and immaturity	Congestion, autolytic changes and immaturity	Congestion, autolytic changes and immaturity	Congestion, autolytic changes and immaturity	Two arteries and one vein in Wharton’s jelly; no pathological findings

Findings of organ immaturity, patent foramen ovale, and patent ductus arteriosus in the preterm cases were considered compatible with the developmental stage and gestational age and were not interpreted as specific effects of methamphetamine exposure.

## Data Availability

The data supporting the findings of this study are available from the corresponding author upon reasonable request. The data are not publicly available due to privacy and ethical restrictions.
